# Phosphoinositide 3 Kinase Signaling in Human Stem Cells from Reprogramming to Differentiation: A Tale in Cytoplasmic and Nuclear Compartments

**DOI:** 10.3390/ijms20082026

**Published:** 2019-04-24

**Authors:** Giulia Ramazzotti, Stefano Ratti, Roberta Fiume, Matilde Yung Follo, Anna Maria Billi, Isabella Rusciano, Eric Owusu Obeng, Lucia Manzoli, Lucio Cocco, Irene Faenza

**Affiliations:** Department of Biomedical Sciences, University of Bologna, Via Irnerio, 48, 40126 Bologna, Italy; giulia.ramazzotti@unibo.it (G.R.); stefano.ratti@unibo.it (S.R.); roberta.fiume@unibo.it (R.F.); matilde.follo@unibo.it (M.Y.F.); annamaria.billi@unibo.it (A.M.B.); isabella.rusciano3@unibo.it (I.R.); eric.owusuobeng2@unibo.it (E.O.O.); lucia.manzoli@unibo.it (L.M.)

**Keywords:** stem cells, nucleus, inositide signaling

## Abstract

Stem cells are undifferentiated cells that can give rise to several different cell types and can self-renew. Given their ability to differentiate into different lineages, stem cells retain huge therapeutic potential for regenerative medicine. Therefore, the understanding of the signaling pathways involved in stem cell pluripotency maintenance and differentiation has a paramount importance in order to understand these biological processes and to develop therapeutic strategies. In this review, we focus on phosphoinositide 3 kinase (PI3K) since its signaling pathway regulates many cellular processes, such as cell growth, proliferation, survival, and cellular transformation. Precisely, in human stem cells, the PI3K cascade is involved in different processes from pluripotency and induced pluripotent stem cell (iPSC) reprogramming to mesenchymal and oral mesenchymal differentiation, through different and interconnected mechanisms.

## 1. Introduction

Stem cells are undifferentiated cells that can self-renew and differentiate into specialized cell types under proper conditions. According to their differentiation potential, stem cells can be classified as pluripotent stem cells, like embryonic stem cells (ESCs) or induced pluripotent stem cells (iPSCs) that can give rise to cells of all three embryonic lineages, or multipotent stem cells, such as mesenchymal stem cells (MSCs), that can differentiate into multiple specialized cells of a specific lineage [1]. Besides, progenitor cells differentiate only into one cell type, like skin stem cells that give rise to keratinocytes.

Stem cells have been widely used successfully for clinical applications, like bone marrow transplantation to treat hematological disorders like leukemia, anemia, and immunodeficiencies, and skin stem cells to heal severe burns. On the other hand, pluripotent stem cells, like ESCs and patient-specific iPSCs, have a terrific therapeutic potential, but several drawbacks still need to be overcome in order to develop successful clinical applications. In fact, in the last decade, stem cell therapy based solely on the administration of stem cells in suspension in order to repair damaged tissue showed limited efficacy in many clinical trials [2,3]. Actually, the lack of appropriate intercellular contacts, nutrition, and regulatory signals caused the failure of stem cell integration in the damaged tissue as they survived only for a few weeks secreting paracrine factors [4,5]. Therefore, the understanding of the signaling pathways involved in stem cell pluripotency maintenance and differentiation is of paramount importance in order to understand these biological processes and to develop therapeutic strategies.

In this review, we focus on phosphoinositide 3-kinase (PI3K) signaling because of its well documented involvement in the control of several cellular processes, such as cell growth, proliferation, survival, and cellular transformation. Different stimuli, including a range of growth factors and mitogens, activate cell surface tyrosine kinase receptors, which in turn determine the activation of PI3K. PI3K is a lipid kinase that upon activation phosphorylates phosphatidylinositol-4,5-bisphosphate (PIP2) to phosphatidylinositol-3,4,5-trisphosphate (PIP3), which sequentially activates pleckstrin homology (PH) domain-containing proteins, such as 3-phosphoinositidedependent kinase-1 (PDK1) and Akt [6]. In order to achieve full activation, Akt is then phosphorylated by mTORC2. PI3K signaling is downregulated by the activity of the lipid phosphatases PTEN and SHIP1/2 that dephosphorylate PIP3 [7,8,9]. Interestingly, PI3K signaling takes place in both cytoplasm and nuclei [10].

## 2. PI3K in Human Embryonic Stem Cell Pluripotency and iPSC Reprogramming

Human embryonic stem cells (hESCs) derive from the inner cell mass of the preimplantation blastocyst. When cultured under proper conditions, these cells can be propagated in vitro for a prolonged time, retain their pluripotency, and can differentiate into all three germ layers. As hESCs are derived from embryos, hESC research poses several ethical concerns that can be bypassed by the development of induced pluripotent stem cells (iPSCs). iPSCs are adult somatic cells reprogrammed into pluripotent cells by the forced expression of transcription factors [11,12].

**Figure 1:**
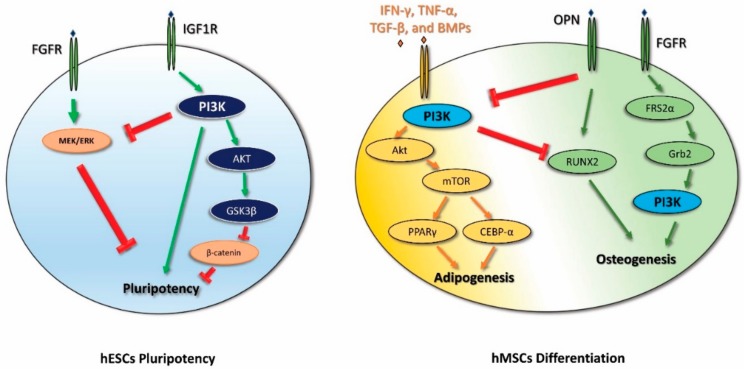
Schematic diagram that outlines phosphoinositide 3 kinase (PI3K) signaling from pluripotency maintenance to differentiation in human stem cells. Green arrows represent activation, red represent inhibition. FGFR, fibroblast growth factor receptor; IGF1R, insulin-like growth factor 1 receptor; OPN, osteopontin.

The PI3K pathway is important for the maintenance of the undifferentiated state of hESCs, as has been demonstrated by several studies through the genetic and pharmacological inhibition approach. Indeed, PI3K inhibition results in the downregulation of pluripotency markers and at the same time in the upregulation of lineage-specific genes, hinting at an overall loss of pluripotency [13,14,15]. The maintenance of the undifferentiated state of hESCs requires the interaction of different signaling pathways. The presence of basic fibroblast growth factor (bFGF) in the culture medium of hESCs determines the activation of EGFR and IGF1R that in turn stimulates both PI3K and MEK/ERK signaling pathways [16]. Initially, the authors showed that both signaling pathways are active in hESC pluripotency upon bFGF stimulation [15]. However, subsequently, the significant role of PI3K/Akt signaling in pluripotency maintenance was highlighted [17], whereas ERK1/2 signaling was shown to be important in controlling endodermal differentiation [18,19]. Furthermore, active PI3K signaling determines the inhibition of the MAPK/ERK pathway and the activation of glycogen synthase kinase-3β (GSK3β). GSK3β in turn downregulates the Wnt/β-catenin pathway, and thus contributes to maintaining the undifferentiated state of hESCs [14,20,21,22]. Moreover, through β-catenin inhibition, PI3K signaling can also affect the Activin A/Smad 2/3 pathway by switching its activity from promoting differentiation to supporting self-renewal [21] (Figure 1).

The role of PI3K/Akt signaling in iPSCs has not been completely investigated yet. It has been reported that the PI3K pathway promotes iPSCs reprogramming by inhibiting GSK3β and forkhead box protein O1 (FOXO1) [23,24], and that Akt inhibition blocks the cell reprogramming process [25]. Moreover, in the early steps of iPSC reprogramming, PI3K signaling is involved in switching from oxidative phosphorylation to glycolysis as it matches the increase in glycolytic gene expression [26,27]. In particular, the allosteric PDK1 activator PS48 supports reprogramming by upregulating glycolytic genes [28]. Moreover, the PI3K/Akt pathway is essential for the survival of iPSCs, as demonstrated by the administration of Wortmannin, an inhibitor of PI3K/Akt signaling that induces apoptosis in iPSCs through the activation of caspase-3.

The role of the CDK1-PDK1-PI3K/Akt signaling pathway has been associated with the regulation of embryonic and induced pluripotency. Specifically, somatic reprogramming regulation is promoted by cyclin B1-CDK1 complexes that are responsible for the maturation and differentiation of iPSCs. Moreover, the monitoring of iPSC factors could be considered a new possibility for the enhancement of reprogramming efficiency [29].

## 3. PI3K in Mesenchymal Stem Cell Differentiation

Human mesenchymal stem cells (hMSCs) can be isolated from different tissues, including adipose tissue, amniotic fluid, bone marrow, endometrium, dental tissues, umbilical cord, and Wharton’s jelly. These cells can differentiate into adipocytes, cardiomyocytes, chondrocytes, and osteocytes, as well as neurocytes and hepatocytes, depending on the microenvironment. Moreover, they can affect the differentiation of host cells through the secretion of cytokines and growth factors, which may enhance the repair of damaged tissues. Therefore, they represent a promising cell source for tissue repair and the treatment of various pathological conditions [30,31].

Several signaling pathways have been shown to play important roles in regulating the adipogenic and osteogenic differentiation of MSCs, including transforming growth factor beta (TGFβ)/bone morphogenic protein (BMP) signaling, Wnt signaling, Hedgehog (Hh) signaling, and Notch signaling [32]. Their activity is also regulated by interaction with other signaling pathways, like fibroblast growth factor (FGF) and platelet-derived growth factor (PDGF) pathways. In particular, the FGF receptor signaling cascade was demonstrated to increase osteogenesis by recruiting the docking protein FRS2α that contains four binding sites for the adaptor protein Grb2, which in turn recruits and activates PI3K [33,34]. Moreover, the PDGF signaling pathway was determined to promote osteogenic differentiation by interacting with the TGFβ signaling pathway. Its signaling activity involves the phosphorylation of PI3K and Akt, as demonstrated by the partial suppression of MSC differentiation by the administration of the PI3K inhibitor LY294002. Besides, PDGF-stimulated and PI3K/Akt-mediated signaling enhances the TGFβ-induced osteogenic differentiation of hMSCs in an MEK/ERK-dependent manner. The combination of PDGF-activated PI3K/Akt and TGFβ-activated MEK pathways promotes osteogenic differentiation [35]. Interestingly, some studies suggested that PDGF receptor signaling, on the contrary, is not associated with hMSC osteogenic differentiation even if it is involved in MSC proliferation [36]. This observation further underlines the importance of continuing a deep investigation of this very intriguing pathway. The PI3K pathway is also involved in the MSC lineage commitment mediated by physical factors. In fact, the adipogenic versus osteogenic balance of MSCs is also regulated by the binding of components of the extra-cellular matrix, like osteopontin and fibronectin, to integrins. Integrins are transmembrane receptors that mediate cell-to-matrix and cell-to-cell interactions. Ligands binding to integrins leads to their activation, which results in the phosphorylation of focal adhesion kinase (FAK) followed by the activation of a series of signaling proteins including PI3K [37].

During adipogenesis, the expression levels of the mediators of the PI3K/Akt pathway and its downstream proteins mTOR, FOXO1, p27 (kip1), and p70S6K are increased. The PI3K inhibitor LY294002 is able to decrease the adipogenic differentiation of MSCs, indicating that the PI3K pathway is important for adipogenesis. Moreover, Lar (leukocyte common antigen-related tyrosine phosphatase) inhibits the PI3K pathway and subsequently reduces the adipogenic differentiation of MSCs [38,39,40]. Other studies showed that the adipogenic differentiation of hMSCs requires a time-dependent modulation of the PI3K/AKT/mTOR pathway in order to promote autophagy-mediated differentiation by blocking Notch signaling [41] (Figure 1).

The PI3k pathway also plays a role in the differentiation of a particular type of MSC, i.e., adipose-derived stem cells (ADSCs). ADSCs display the multipotent characteristic of MSCs and have the advantage of being obtained from abundant adipose tissue with a minimally invasive procedure, resulting in a high number of cells. Hence, ADSCs represent a promising tool for regenerative medicine.

During adipogenic differentiation of ADSCs, the PI3K/Akt signaling pathway is strongly activated, whereas its inhibition by Wortmannin, an Akt inhibitor, decreases adipogenesis [42]. Moreover, adipogenesis is also stimulated by hypoxia, which induces differentiation via mitochondrial ROS generation and activates the PI3K/Akt pathway [43].

Besides, the PI3K/Akt signaling pathway promotes osteogenic differentiation of ADSCs and it takes part in a positive feedback loop that involves interleukin 6 (IL6) and its receptor. In fact, during osteogenesis, IL6 activates the PI3K/Akt pathway, which in turn increases IL6-receptor expression, further promoting the process [44].

It is worth noting that another lipid signaling enzyme that uses PIP2 as a substrate like PI3K is involved in osteogenesis in ADSCs, i.e., phospholipase C-β1 (PLC-β1). PLC-β1 hydrolyses PIP2 to produce two second messengers: inositol 1,4,5-trisphosphate (IP3) and diacylglycerol (DAG), which in turn modulate many downstream effectors. The expression of PLC-β1 is required for osteogenic differentiation, as its silencing inhibits the differentiation process, and it is time-dependent, as its levels increase during the later stages of differentiation [45,46]. Moreover, inositide signaling in the nucleus plays an important role in both normal hematopoiesis and myelodysplastic syndromes [47]. Indeed, the role of phospholipid metabolism and of PLC-β1 activity in both cytoplasmic and nuclear compartments has been deeply investigated in many signaling processes involved in differentiation, proliferation, and cell cycle regulation of several experimental models [48,49,50,51].

An interesting feature of ADSCs is their ability to secrete exosomes. Exosomes are 30- to 150-nm-sized nanoparticles produced from multivesicular bodies and are important paracrine effectors in intercellular communication. They can transfer proteins and genetic materials to target cells [52]. Exosomes show functional properties similar to those of the cells from which they originate with no apparent adverse effects [53,54]. Notably, ADSC-derived exosomes are potential players in the regeneration and protection of several tissues, including skin, bone, muscle, and brain tissue [55,56,57,58].

ADSC-derived exosomes are able to enhance the growth of skeletal muscle and Schwann cell lines in a dose-dependent manner. Proteomics analysis of their content showed that they enclose proteins involved in different signaling pathways related to skeletal muscle and nerve regeneration and proliferation, including proteins associated with the PI3K/Akt pathway [59].

Moreover, ADSC-derived exosomes can speed up the wound-healing process in a mouse model in vivo and activate the PI3K/Akt signaling pathway both in vivo and in vitro. In fact, upon exosome treatment, fibroblasts show a significant and dose-dependent increase in cell proliferation and migration. These exosome-induced changes are reduced by the administration of the PI3K inhibitor LY294002, suggesting that the exosomes’ ability to promote collagen deposition and further stimulate wound healing requires the activation of the PI3K/Akt signaling pathway [60].

## 4. PI3K in Oral Mesenchymal Stem Cell Differentiation

MSCs have also been identified in many perioral tissues such as dental pulp, periodontal ligaments, dental follicles (DFPCs), gingival tissue (GMSCs), alveolar bone, apical papilla (SCAP), and deciduous teeth (SHED). They all present in vitro multipotency and the ability to differentiate into odontoblasts, osteocytes, adipocytes, chondrocytes, neural cells fibroblasts, and endothelial cells [61].

Human dental pulp stem cells (hDPSCs), as well as human periodontal ligament stem cells (hPDLSCs), are ectodermal-derived stem cells that originate from migrating neural crest cells. Compared to bone marrow MSCs, hDPSCs show a higher proliferation rate and higher clonogenic and mineralization potential [62].

During dental pulp inflammation, hDPSCs can contribute to the repair of damage resulting from the late stages of dental caries that cause apoptosis of odontoblasts. hDPSCs are found in the stem cell niche around the blood vessel, hence in order to take part in dental regeneration, i.e., the regeneration of odontoblasts and the formation of reparative dentin, they have to migrate to the site of damage [63]. Their migration is stimulated by chemokines expressed in the extracellular matrix, like stromal cell-derived factor-1 (SDF-1). Through its binding to CXCR4 (C-X-C chemokine receptor 4), SDF-1 is able to induce the concentration-dependent migration of hDPSCs. It controls the phosphorylation of FAK (focal adhesion kinases) on cell membranes and the translocation of β-catenin to the nucleus. This process involves PI3K, Akt and GSK3β phosphorylation [64,65].

**Figure 2:**
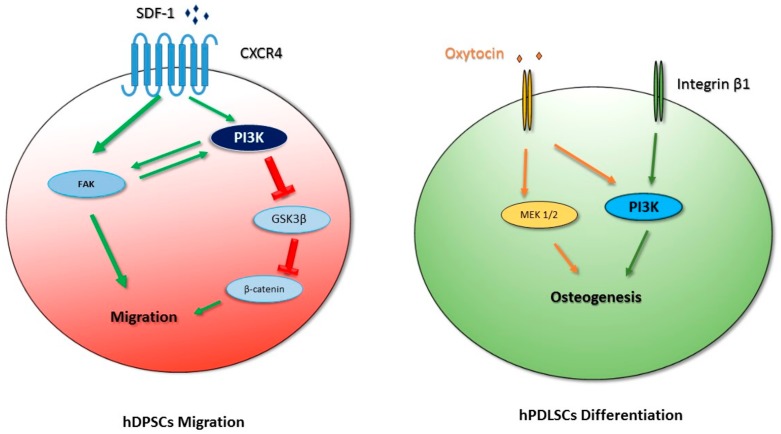
Schematic diagram that outlines PI3K signaling in human oral stem cells. Green arrows represent activation, red represent inhibition.

Chemical inhibitors and RNA silencing approaches indicated that the effect of SDF-1 on the migration of hDPSCs depends on PI3K/Akt signaling, since inhibition of the PI3K pathway remarkably reduces the expression of β-catenin and the phosphorylation of Akt and GSK3β, suggesting that they are the downstream effectors of SDF-1/CXCR4/PI3K signaling. Hence, PI3K/Akt and GSK3β/β-catenin pathways are involved in the control of hDPSC migration [64] (Figure 2).

Moreover, PI3K signaling is involved in the response to physiological hypoxia in hDPSCs in vitro. Physiological hypoxia causes an upregulation of cellular glycolytic metabolism and a decrease in the activity of the tricarboxylic acid cycle and oxidative phosphorylation, which determines a reduction in mitochondrial ROS production [66,67]. ROS can act both as signaling molecules or as harmful factors associated with high levels of tissue damage [66]. Many studies have reported that the PI3K/Akt pathway affects intracellular ROS production and can regulate the pathways involved in oxidative stress. Their downstream effectors are FOXO1 and caspase-3, whose expression is decreased in response to PI3K signaling [68,69,70]. Besides, the PI3K/Akt pathway controls the translocation of hypoxia-inducible factor 1-alpha (HIF-1α) to the nucleus, since Akt inhibition causes inhibition in HIF-1α translocation [71].

Besides, PDLSCs represent one of the most promising sources of stem cells for periodontal regenerative medicine due to their ability to differentiate into osteogenic, adipogenic, and chondrogenic cells in vitro and into bone-, PDL-, and cementum-forming lineages in animal models in vivo [72,73,74,75].

As it has been shown for MSCs from different tissues, the PI3K/Akt pathway is involved in the osteogenic differentiation of hPDLSCs in vitro. However, the different signaling pathways involved, like PI3K/Akt, MAPK/ERK, and p38 MAPK, display different levels of activation in different MSCs after osteoblast induction [76]. In hPDLSCs, osteogenesis can be promoted by different stimuli. For example, oxytocin can induce osteogenic differentiation of PDLSCs through the activation of ERK and Akt pathways [77] (Figure 2).

An important issue to solve in order to exploit hPDLSCs for tissue regeneration in vivo is the reduction of their pluripotency in an inflammatory environment, since this loss of functionality leads to an impaired regenerative potential and immunomodulatory effect. It has been shown that the receptor P2X7 plays an important role in inducing hPDLSC osteogenesis and mineralization under inflammatory conditions. P2X7R overexpression causes an increase in mTOR expression and in PI3K, Akt, and mTOR phosphorylation, whereas blocking P2X7R activation has the opposite effect. Moreover, suppression of PI3K/Akt signaling inhibits P2X7R-induced osteogenesis [78].

The PI3K pathway is also involved in osteogenic differentiation induced by mechanical stimuli, such as the activation of integrin α5/β1 by binding to a bioadhesive substrate. In fact, it has been demonstrated that the isoform p110γ of PI3K interacts with integrin β1, and this interaction determines PI3K activation and the consequent induction of osteogenesis [79] (Figure 2).

hPDLSCs can also differentiate into neural cells. During the differentiation process, the cells show a prolonged survival and inhibition of apoptosis, which is associated with an increase in the activation of the PI3K/Akt pathway [80]. The neuronal commitment of hPDLSCs also involves the activity of protein kinase C α (PKCα), which is a downstream effector of lipid signaling pathways. In fact, during neurogenic commitment, PKCα is phosphorylated and translocates to the nucleus-inducing growth associated protein-43 (GAP-43) phosphorylation and its export to the cytosol with a consequent accumulation at the cell periphery, where it regulates growth cone dynamics and neuronal differentiation [81].

## 5. Conclusions

PI3K signaling regulates many cellular processes, such as cell growth, proliferation, survival, and cellular transformation. Specifically, the PI3K cascade is involved in different processes of human stem cells, from pluripotency and iPSC reprogramming to mesenchymal and oral mesenchymal differentiation, through different and interconnected mechanisms:

- *iPSC reprogramming*: PI3K seems to promote iPSC reprogramming by inhibiting GSK3β and FOXO1. Akt inhibition stops the reprogramming process of the cells, and, in the early steps of iPSC reprogramming, PI3K is involved in the switching from oxidative phosphorylation to glycolysis. The PI3K/Akt pathway also plays a pivotal role in the survival of iPSCs. The administration of Wortmannin, an inhibitor of PI3K/Akt signaling, induces apoptosis in iPSCs through caspase-3 activation.
- *Adipogenic and osteogenic mesenchymal differentiation*: During adipogenesis, the PI3K/Akt pathway is activated and its downstream mediators mTOR, FOXO1, p27, and p70S6K are increased. Indeed, the administration of the PI3K inhibitor LY294002 decreases the adipogenic differentiation of MSCs. Moreover, adipogenic differentiation requires a time-dependent modulation of the PI3K/Akt/mTOR pathway in order to promote autophagy-mediated differentiation. The PI3K pathway is involved in osteogenic differentiation as LY294002 partially suppresses this process, and it is associated with the MSC lineage commitment mediated by physical factors. Furthermore, the PI3k pathway promotes osteogenic differentiation of a particular type of MSC—ADSCs.
- *Oral mesenchymal differentiation*: In hDPSCs, the PI3K pathway is involved in the control of migration and it affects the expression of β-catenin and the phosphorylation of Akt and GSK3β. In addition, PI3K is involved in the response to physiological hypoxia in hDPSCs in vitro, where it controls intracellular ROS production and regulates oxidative stress. The PI3K/Akt pathway also controls the translocation of HIF-1α to the nucleus, since Akt inhibition causes inhibition in HIF-1α translocation. In PDLSCs, the PI3K/Akt pathway is involved in osteogenic differentiation promoted by different stimuli. For example, in osteogenic differentiation induced by mechanical stimuli, such as the activation of integrin α5/β1 by binding to a bioadhesive substrate, the isoform p110γ of PI3K interacts with integrin β1, and this interaction determines PI3K activation and the consequent induction of osteogenesis. Moreover, hPDLSCs can also differentiate into neural cells.

Overall, the above information underlines the role of PI3K signaling in iPSCs reprogramming and several mesenchymal differentiation processes. Therefore, the understanding of PI3K signaling in human stem cells is still challenging, but it might pave the way to the understanding of different physiopathological mechanisms and to new and promising approaches in regenerative therapies.
